# Cellular Antioxidant Effect of Four Bromophenols from the Red Algae, *Vertebrata lanosa*

**DOI:** 10.3390/md11082769

**Published:** 2013-08-05

**Authors:** Elisabeth K. Olsen, Espen Hansen, Johan Isaksson, Jeanette H. Andersen

**Affiliations:** 1 MabCent-SFI, University of Tromsø, Breivika, N-9037, Tromsø, Norway; 2 Marbio, University of Tromsø, Breivika, N-9037, Tromsø, Norway; E-Mails: espen.hansen@uit.no (E.H.); jeanette.h.andersen@uit.no (J.H.A.); 3 Department of Chemistry, University of Tromsø, Breivika, N-9037 Tromsø, Norway; E-Mail: johan.isaksson@uit.no

**Keywords:** marine algae, macro algae, bioactivity, cellular antioxidant assay, ORAC

## Abstract

Three known bromophenols, 2,3-dibromo-4,5-dihydroxybenzylaldehyde (**1**), 2,2′,3-tribromo-3′,4,4′,5-tetrahydroxy-6′-hydroxymethyldiphenylmethane (**2**) and bis(2,3-dibromo-4,5-dihydroxylbenzyl) ether (**3**), and one new one, 5,5″-oxybis(methylene)bis(3-bromo-4-(2′,3′-dibromo-4′,5′-dihydroxylbenzyl)benzene-1,2-diol) (**4**), were isolated from an extract of the red alga, *Vertebrata lanosa*. The antioxidant activity of these four bromophenols was examined using one biochemical and two cellular assays: Oxygen Radical Absorbance Capacity (ORAC), Cellular Antioxidant Activity (CAA) and Cellular Lipid Peroxidation Antioxidant Activity (CLPAA) assays. Compound **2** distinguished itself by showing potent activity, having a better antioxidant effect than luteolin in both the CAA and CLPAA assays and of quercetin in the CLPAA assay. Although several bromophenols are known to be potent antioxidants in biochemical assays, this is the first time their cellular antioxidant activity has been demonstrated.

## 1. Introduction

Marine macroalgae are known to contain structurally diverse natural compounds with a range of different biological activities [1]. One class of compounds derived from macroalgae are known as bromophenols (BPs). The BPs are biosynthesized from polyphenols by bromoperoxidases in the presence of hydrogen peroxide and bromide [2]. They are common marine secondary metabolites [3], although the algal content seems to vary with season [4] and tide [2]. Different biological activities have been reported for the BPs; among these are antioxidant, antimicrobial, anticancer, anti-diabetic and anti-thrombotic effects, which make them interesting compounds in the development of new pharmaceutical agents [3]. 

Structurally, the BPs contain one or several benzene rings and a varying number of bromine and hydroxyl-substituents [3]. The relationship between the molecular structure of some BPs and their antioxidant effect have previously been investigated [5], and the trend is that the activity is improved by both an increased number of hydroxyl groups and conjugations [6,7,8]. According to the review of Liu and co-workers, the 1,4-dihydroxy arrangement seems to be favorable for antioxidant activity, while bromination appears to be of little importance [3]. When natural BPs were compared to their corresponding debrominated compounds, it was discovered that bromination even decreased the antioxidant effect [9]. 

The first marine BP was isolated from the red alga *Polysiphonia morrowii* in 1955 [10], and subsequently several BPs have been isolated and identified from red, brown and green algae [3]. Some species of red algae have concentrations of BPs up to 2590 ng/g [2], and more than 30 monoaryl and diaryl BPs have been discovered in the *Rhodomelaceae* family [11]. Marine alga is not the only marine source of these metabolites, as they are also found in ascidians and sponges [3]. The red alga *Vertebrata lanosa* is a member of the family *Rhodomelaceae.* Several synonyms are known for this alga, the name *Polysiphonia lanosa* has also been widely used. The alga is commonly distributed in Europe, and it is also found in North America, Canada, tropical and subtropical Western Atlantic [12]. Crude extracts of *V. lanosa* have been reported to show high antioxidant activity in biochemical assays in addition to having a high phenolic content [13]. 

Antioxidants serve as a defense against free radicals, such as reactive oxygen species (ROS) and reactive nitrogen species (RNS). ROS and RNS form naturally during many metabolic processes, when well regulated, they contribute toward maintaining homeostasis in normal healthy cells and work as signaling molecules [14]. However, the level of free radicals can increase if this balance is lost, which can happen in response to xenobiotics or environmental stress. When the balance is shifted towards pro-oxidants, a state of oxidative stress occurs, this condition can be a contributing factor to the development of several medical conditions, such as cardiovascular diseases, including atherosclerosis, various types of cancer, diabetes and neurodegenerative diseases, like Parkinson’s and Alzheimer’s disease. Cells have several protective mechanisms against the harmful effects of ROS and RNS, both enzymatic (e.g., superoxide dismutases, catalase and glutathione peroxidase) and nonenzymatic (e.g., GSH, NADPH, α-tocopherol and ascorbic acid) [14,15,16]. These antioxidant mechanisms work to prevent, intercept and repair the damage caused by the free radicals [14].

Recently, a study was published where the antioxidant activities of 19 naturally occurring BPs were reported, six of which were new, from the alga *Rhodomela confervoides*. Most of the isolated compounds showed a significant antioxidant activity against 2,2-Diphenyl-1-picrylhydrazyl (DPPH) and 2,2′-Azino-bis(3-ethylbenzothiazoline-6-sulfonic acid) (ABTS) radicals [5]. At present, approximately 30 BPs with antioxidant activity have been reported from marine algae. The published studies have only investigated the activity *in vitro*, mainly determined by the DPPH radical scavenging method [3]. 

Thus, the purpose of the present study was to further examine the antioxidant potential of one new and three known BPs isolated form *V. lanosa*. In addition to a biochemical assay, Oxygen Radical Absorbance Capacity (ORAC), two cellular assays were included; Cellular Antioxidant Activity (CAA) assay and Cellular Lipid Peroxidation Antioxidant Activity (CLPAA) assay. The two latter methods allowed us to explore the bioavailability of the compounds, particularly if they were able to pass cellular membranes. 

## 2. Results and Discussion

### 2.1. Isolation and Characterization

**Scheme 1:**
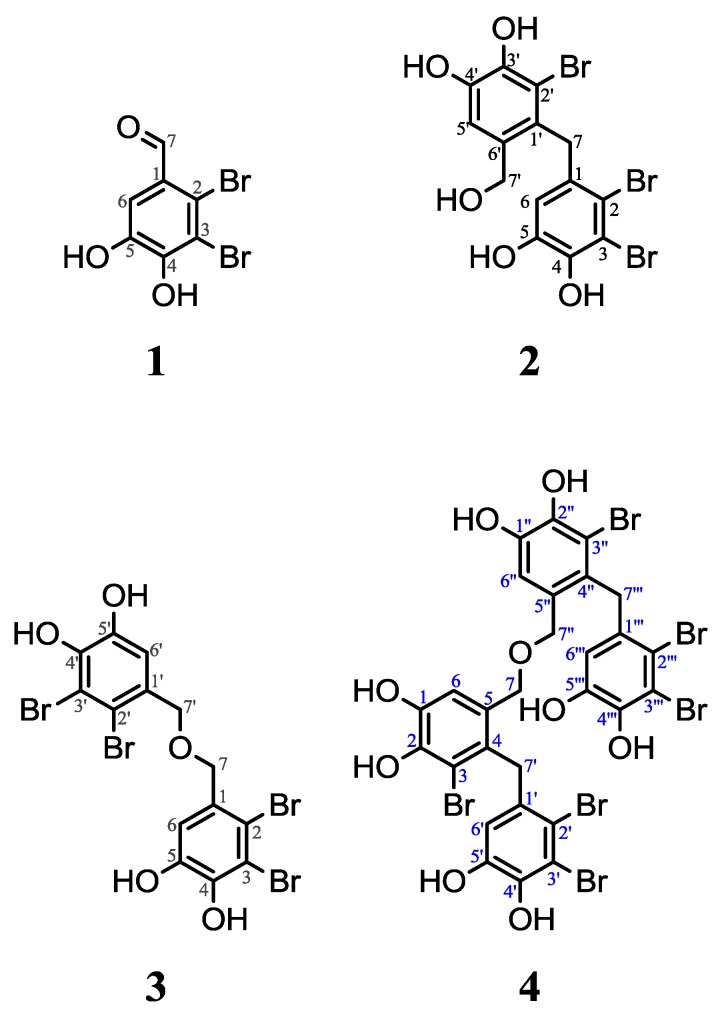
Molecular structures of compounds **1**–**4**. Proton and carbon denotations are given (color codes are used for compounds **2** (black) and **4** (blue) to distinguish them in the NMR spectra (Figures S2 to S5 in the Supplementary Material)).

Compounds **1**–**4** (Scheme 1) were all isolated from the red alga *V. lanosa* collected in Oldervik, Ullsfjorden, Norway. Bioactivity screening of the algal extract indicated both anticancer and antioxidant effects. Brominated compounds were isolated by mass guided preparative HPLC, followed by structure characterization of compounds **1**–**4** using high resolution MS and NMR. 

### 2.2. Identification of Compound **4**

The NMR spectra of compound **4** was found to be near identical to those of the previously reported compound **2**, with the exception of a downfield shift of C6 of approximately 10 ppm and an apparent long range ^3^*J*_CH_ from C6 to the directly bound H6 in the HMBC spectrum (see Figures S2 to S5 in the Supporting Material). The superimposed HSQC and HMBC spectra display overlapping correlations in both HSQC and HMBC for C6H6. This becomes possible only if compound **4** is the symmetric dimer of compound **2**, allowing the ^3^*J*_C6H6'_ and ^3^*J*_C6'H6_ couplings to form a correlation in the ^13^C-HMBC, perfectly matching the ^1^*J*_C6H6_ and ^1^*J*_C6'H6'_ correlations in the ^13^C-HSQC. All ^13^C and ^1^H chemical shifts, in addition to the observed HMBC correlations of compound **4** (Scheme 1), are summarized in Table 1. 

**Table 1:** NMR spectroscopic data (600 MHz, methanol-*d*_4_) for compounds **2** and **4** referenced against the solvent residual peak for methanol-*d*_4_: ^1^H = 3.31 ppm, ^13^C = 49.00 ppm. For proton and carbon denotations, see Scheme 1.

	compound 2			compound 4			
#	δ_C_, type	δ_H_	HMBC (H → C) ^a^	#	δ_C_, type	δ_H_	HMBC (H → C) ^a^
1	132.62, C			1′″	132.82, C		
2	116.29, C			2′″	116.39, C		
3	114.19, C			3′″	114.24, C		
4	143.76, C			4′″	143.68, C	6.84,s	2, 3, 6, 7
5	146.20, C			5′″	146.08, C		
6	115.03, CH	6.07,s	8, 9, 11, 12, 14	6′″	115.16, CH	6.03,s	8, 9, 11, 12, 14
7	39.65, CH_2_	4.13,s	5, 7, 9, 10, 11, 12, 14	7′″	39.82, CH_2_	4.05,s	5, 7, 9, 10, 11, 12, 14
1′	128.97, C			4″	130.64, C		4, 5, 6', 7
2′	115.49, C			3″	116.20, C		
3′	143.76, C			2″	144.44, C		8, 9, 11, 12, 14
4′	145.82, C			1″	145.54, C		
5′	115.55, CH	6.96,s	2, 3, 6, 7	6″	117.25, CH	6.84,s	2, 3, 6, 7
6′	133.48, C			5″	130.17, C		
7′	63.21, CH_2_	4.36,s	4, 5, 7	7″	71.94, CH_2_	4.20,s	4, 5, 6', 7

^a^ HMBC correlations, optimized for 8 Hz, are from proton(s) to the indicated carbon.

Compound **4** has not previously been reported. However, we cannot exclude that it might be an artefact of the extraction and purification process. In ^1^H NMR spectra for compound **2**, it was observed that compound **4** formed gradually during isolation, from 0.17 mol% in the beginning to 0.25 mol% at the end. In the presence of acid and water, as in a protonated aqueous mixture after purification by HPLC, dimerization of compound **2** might occur. Dehydration of benzyl alcohol to dibenzyl ether has previously been reported [17]. Benzyl alcohol is less sterically hindered than compound **2** and does not contain any substituents on the aromatic ring. Nevertheless, the data presented indicates that condensation between two aromatic compounds containing primary alcohols is possible. An experiment was performed, where compound **2** was dissolved in H_2_O with 0.1% formic acid to imitate the conditions after HPLC. ^1^H NMR data were acquired before and after storage at 4 °C for seven days. Peaks corresponding to protons on carbon 5′, 6, 7′ and 7 (for compound **2**) and (6,6″), (6′,6′″), (7,7″) and (7′,7′″) (for compound **4**) were used to examine if a dimerization had occurred. When ratios of peak intensities between compounds **2** and **4** were compared, an alteration from 3.6:1 mol% to 2.6:1 mol% was found after seven days of storage. This demonstrates that compound **2** is acid labile and can convert to compound **4** upon prolonged storage in acidic water. Hence, compound **2** should not be kept in a protonated aqueous environment for a long time, as dimerization reduces the antioxidant effect. 

### 2.3. Antioxidant Activity

The mechanisms behind antioxidant protection of cells are many and complex; they can be both enzymatic and non-enzymatic, one single assay cannot reflect the complex interactions that occur between antioxidants and their targets *in vivo* [18]. It is therefore necessary to apply several complimentary assays in order to assess the biological relevance of an antioxidant. In this study, we used the ORAC assay to see if the compounds had antioxidant activity in general and the cellular assays CAA and CLPAA were included to get complimentary information about bioavailability. 

#### 2.3.1. Antioxidant Assay for Oxygen Radical Absorbance Capacity (ORAC)

This biochemical assay measures the oxidative degeneration of fluorescein and is based on the procedure by Huang *et al.* [19]. Antioxidants are able to protect fluorescein from degradation after exposure to 2,2′-azobis(2-methylpropioanamidine) dihydrochloride (AAPH) radicals, and this results in a reduction of fluorescence. 

Quercetin and luteolin are known to have antioxidant activity and are commonly used as reference compounds. Wolfe and co-workers classifies them as having a moderate effect in the ORAC assay [20], it has also been reported by our research group [21]. When examined in this study, quercetin and luteolin displayed activities of 7 and 10 µM Trolox equivalents (TE), respectively, at 1 μg/mL. 

**Figure 1:**
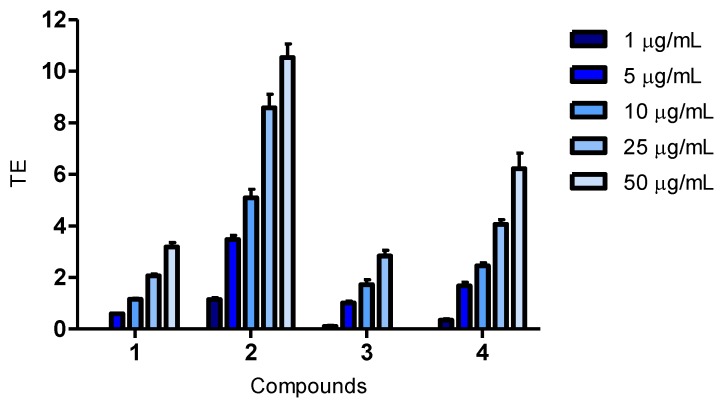
Oxygen Radical Absorbance Capacity (ORAC): oxidative degeneration of fluorescein after the addition of 2,2′-azobis(2-methylpropioanamidine) dihydrochloride (AAPH), measured in μM trolox equivalents. Compounds **1**–**4** were examined in concentrations of 1, 5, 10, 25 and 50 μg/mL. The bars indicate mean Trolox equivalents (TE) with SEM; *n* = 4 from two independent runs. The highest concentration tested for compound **3** was 25 µg/mL.

All of the compounds showed a dose-dependent activity. The antioxidant activity was found to be highest for compound **2**, followed by compound **4**, as seen in Figure 1. Compounds **1** and **3** were found to be the least active in the assay. Although compound **2** was the most active of the compounds isolated, it was not as active as quercetin and luteolin. 

#### 2.3.2. Antioxidant Assay for Cellular Antioxidant Activity (CAA)

The assay was performed based on the procedure of Wolfe and Liu (2007) [22]. 

Compounds found active in the CAA assay are intracellular antioxidants. They have the ability to penetrate the cell membrane and reduce the oxidation of 2′7′-dichlorofluorescin (DCFH), a reaction activated using a radical initiator, like AAPH, to the fluorescent molecule, DCF. 

**Figure 2:**
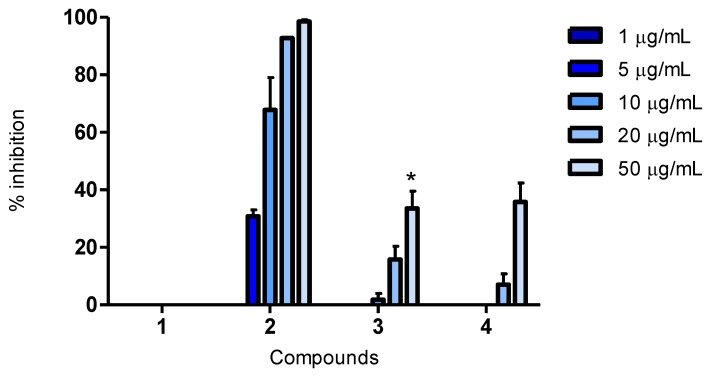
Cellular Antioxidant Activity (CAA): percent inhibition of oxidative degeneration of 2′7′-dichlorofluorescin (DCFH) to DCF inside HepG2 cells after the addition of AAPH and compounds **1**–**4**. Concentrations of 1, 5, 10, 20 and 50 µg/mL were tested. The bars indicate mean percent inhibition, with SEM; *n* = 4 from two independent runs.

In this assay, quercetin and luteolin were able to inhibit the oxidation of DCFH, by 92% and 58%, respectively, at 10 µg/mL. These two known antioxidants have previously been reported to have a high antioxidant activity in the CAA assay, both by our research group and others [20,21]. As seen by Figure 2, compound **2** shows a dose-dependent activity, capable of inhibiting oxidation at concentrations as low as 10 µg/mL (68%). Thus, compound **2** has a significantly better effect than luteolin but was less active than quercetin at the same concentration, making it a promising intracellular antioxidant. Compounds **3** and **4** exhibited a significant inhibition at the highest concentration, while compound **1** did not show activity at any of the concentrations tested (Figure 2).

* The highest concentration tested was 30 μg/mL, not 50 μg/mL.

#### 2.3.3. Cellular Lipid Peroxidation Antioxidant Activity (CLPAA) Assay

The assay was performed based on the procedure of Pap *et al.* [23]. It is used to quantify lipid peroxidation within cell membranes and, to a lesser extent, the plasma membrane. A C_11_-BODIPY probe is distributed heterogeneously throughout cellular membranes and changes from red to green when oxidized, a reaction activated using a radical initiator, like cumene hydroperoxide (cumOOH), and the antioxidant activity is measured as an increase of green fluorescence [24]. 

**Figure 3:**
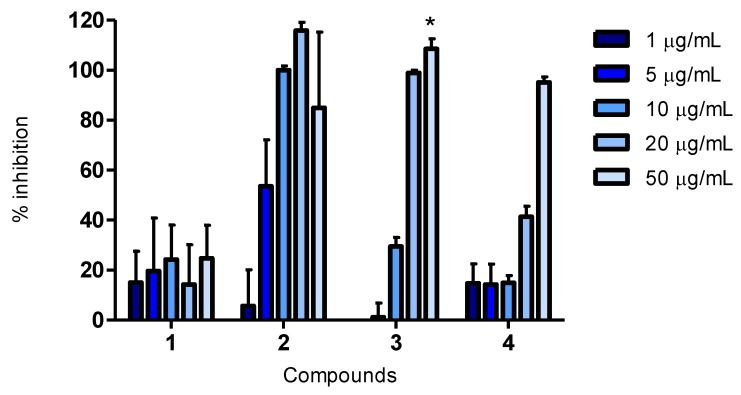
Cellular Lipid Peroxidation Antioxidant Activity (CLPAA): percent inhibition of oxidative degeneration of the C_11_-BODIPY probe in HepG2 cells after the addition of cumene hydroperoxide (cumOOH) and compounds **1**–**4**. Concentrations of 1, 5, 10, 20 and 50 μg/mL were tested. The bars indicate mean percent inhibition with SEM; *n* = 4 from two independent runs.

Quercetin and luteolin gave inhibitions of 35% and 34%, respectively, at 5 μg/mL in this assay. At the same concentration, compound **2** was able to prevent oxidation of the probe at 45%, as illustrated in Figure 3. Thus, the ability to prevent lipid peroxidation within the cell membranes was found to be greater for compound **2** than the known antioxidants in this assay.

Compounds **3** and **4** also showed antioxidant activity, at the two highest concentrations of the former and at the highest concentration of the latter. Compound **1** had a weak activity in the CLPAA assay compared to quercetin and luteolin. 

* The highest concentration tested was 30 μg/mL, not 50 μg/mL.

### 2.4. Cytotoxicity

Cytotoxicity was examined with a viability assay using a cell line of normal human lung fibroblasts (MRC-5). The compounds were tested at 5, 25 and 50 μg/mL. Compound **3** was toxic at 25 μg/mL, and it was concluded that the highest concentration to be used in the cellular antioxidant assays should be 30 μg/mL for compound **3 ** (data not shown). Compounds **1**, **2** and **4** did not show cytotoxicity at the concentrations tested. 

### 2.5. Advantages of Complementing Biochemical Assays with Cellular Ones

Biochemical assays are commonly used to examine antioxidant activity. As noted in the review of Liu and co-workers, antioxidant activity is usually determined by the DPPH radical scavenging method [3]. This is a fast and easy way to determine antioxidant activity, but does not take into account the complex environment of cells, like bioavailability and membrane permeability. 

The use of cellular assays in addition to biochemical ones have also previously been utilized by our research group [21]. Information obtained by the cellular assays is advantageous, as biological systems are much more complex than chemical mixtures. Cellular assays detect only the antioxidants that can penetrate the cell membrane of living cells and inhibit oxidation within the cells. Hence, these assays give additional information on bioavailability, uptake, metabolism and interactions with cellular components, which is more realistic compared to the situation *in vivo* [25]. When including the CAA and CLPAA assays in our study, we gained supplementary information regarding the pharmacokinetic properties of compound **2**, which was found to be able to penetrate the cell membranes and work as an intracellular antioxidant, in addition to preventing lipid peroxidation within cell membranes. 

Antioxidant activity in biochemical and cellular assays are not always equivalent. A study comparing antioxidant activities of flavonoids between the ORAC and CAA assays did not find a significant correlation. Although quercetin and luteolin had moderate activity in the ORAC assay, they both showed a high cellular antioxidant activity. It was concluded that the absence of correlation probably was due to the biological mechanisms of the CAA assay [20]. We found the same to be true for compound **2**, as it was moderately active in the ORAC assay, while displaying a high activity in the CAA assay. 

### 2.6. Assessment of Purity

Compound **2** was not pure when examined in the assays, but existed in a mixture with compound **4** in the ratio of 4:1. Purification proved to be difficult, due to the dimerization of compound **2** into compound **4**, as shown by the experiment of storing the former in acidic water for one week (see Section 2.2). Although not completely pure, we conclude that the antioxidant effect of compound **2** is genuine based on compound **4** displaying a poorer antioxidant effect. Due to this impurity, the actual concentrations of compound **2** examined are likely to be lower than presented here. Thus, compound **2** is possibly effective at even lower concentrations. 

When stored in methanol, the methyl ether of compound **2** was observed in the ^1^H NMR spectrum. A similar incident has also previously been reported by another research group [26]. The methyl ether of compound **2** was removed in the following isolation. 

## 3. Experimental Section

### 3.1. Material

Compounds **1**–**4** were all isolated from the red alga *Vertebrata lanosa*, collected by the marine biobank Marbank in Oldervik, Ullsfjorden (Norway, spring 2010)*.* The alga was freeze-dried and ground before extraction with water, followed by extraction of the remaining organic material using dichloromethane:MeOH 1:1. The extracts were stored at −24 °C until usage. Only the organic extract was used, due to a larger content of the target compounds. 

### 3.2. Sample Preparation

The extract was dissolved in hexane and partitioned twice against MeOH at room temperature. The MeOH phase was reduced to dryness *in vacuo* at 40 °C, resuspended in purified water and partitioned twice against EtOAc at room temperature, followed by the removal of volatiles *in vacuo* at 40 °C and SpeedVac. The EtOAc phase contained a larger concentration of the target compounds and was, thus, used further in the purification process.

### 3.3. Purification

HPLC purification was performed using a Waters (Milford, MA, USA) purification system controlled by MassLynx version 4.1. The purification system consisted of a Waters 600 pump, a Waters 3100 mass spectrometer (in negative mode, with an ESI-electrospray source), a Waters 2996 photo diode array detector and a Waters 2767 sample manager. Samples were injected in 1:1 H_2_O/can, and fraction collection was triggered when the intensity of the target mass exceeded the threshold specified in the method. The compounds were isolated on a Waters XTerra MS-C18 (10 × 250 mm, 5 µm) column and a Waters XSELECT CSH (10 × 250 mm, 5 µm) column. Gradients of H_2_O with 0.1% FA (A) and acetonitrile with 0.1% FA (B) were used at a flow rate of 6 mL/min and optimized for each compound (compound **1**: 50%–55% B over 10 min; compounds **2** and **4**: 50%–60% B over 10 min; compound **3**: 65%–85% B over 25 min). 

### 3.4. Accurate Mass Determination and Elemental Composition of Compounds 1–4 Using High Resolution ESI-MS

Accurate mass determination was performed using a Waters (Milford, MA, USA) UPLC-ToF-MS system controlled by MassLynx version 4.1. The UPLC-ToF-MS system consisted of a Waters LCT Premier and a Waters Acquity UPLC. The compounds were separated on an Acquity UPLC^®^ BEH C_18_ (2.1 × 50 mm, 1.7 μm) column. Gradients of H_2_O with 0.1% FA (A) and acetonitrile with 0.1% FA (B) were used at a flow rate of 0.350 mL/min (20%–100% B over 3.5 min).

Possible elemental compositions of compounds **1**–**4** were calculated using accurate MS, while ChemDraw Pro V 12.0.2 was used to determine the calculated accurate masses. 

The calculated elemental compositions were compared with the literature and the information used in combination with NMR data to elucidate the structures of compounds **1**–**4**. 

Compound **1**: *m/z* 292.8448 [M − H]^+^, calculated for C_7_H_4_Br_2_O_3_ 292.8448. Compound **2**: *m/z* 494.8067 [M − H]^+^, calculated for C_14_H_11_Br_3_O_5_ 494.8078. Compound **3**: *m/z* 572.7182 [M − H]^+^, calculated for C_14_H_10_Br_4_O_5_ 571.7103. Compound **4**: *m/z* 972.6160 [M − H]^+^, calculated for C_28_H_20_Br_6_O_9_ 972.6129.

### 3.5. Identification of Compounds **1**–**4** by NMR

NMR data were used for structure confirmation of compounds **1**–**3** and elucidation of compound **4**. The NMR experiments were acquired on an Agilent (Varian) Inova spectrometer operating at 599.934 and 150.863 MHz for ^1^H and ^13^C, respectively, using a cryogenically cooled inverse detection HCN probe with enhanced proton channel (2nd generation). Typically, 16k data points were acquired for the 1D ^1^H experiments using 90° reading pulses, 5 seconds of relaxation delay and 64 transients. Data matrices of 1440 × 200 and 1440 × 256 complex points were acquired for the gradient selected edited HSQC and absolute value HMBC, respectively, using 32 transients. For carbon 1D spectra, 32k complex data points were acquired, using 45° reading pulses, 1-second relaxation delay and 20,000 transients, applying ^1^H decoupling during both relaxation and acquisition time. All spectra were acquired at 298 K in methanol-*d*_4_ or CDCl_3_ (Sigma-Aldrich) using sweep widths of 9600 and 30,200–37,800 Hz for ^1^H and ^13^C, respectively. All experiments were referenced to the residual solvent peak; 3.34 ppm for methanol-*d*_4_ and 7.26 ppm for CDCl_3_ [27]. 

Compound **1** was identified as 2,3-dibromo-4,5-dihydroxybenzylaldehyde, published for the first time by Katsui and co-workers [28]. ^1^H NMR (600 MHz, methanol-*d*_4_) δ_H_ 7.34 (s, 1H), 10.12 (s, 1H). ^13^C NMR (600 MHz, methanol-*d*_4_) δ_C_ n/a (s, C-3), 152.70 (s, C-4), 146.37 (s, C-5), 114.02 (d, C-6), 126.81 (s, C-1), 192.38 (d, C-7), 122.04 (s, C-2). Impurities of other related compounds (15 mol%) and lipids (25–50 mol%) were present. 

Compound **2** was identified as 2,2′,3-tribromo-3′,4,4′,5-tetrahydroxy-6′-hydroxymethyldiphenylmethane, published for the first time by Fan and co-workers [26]. For ^1^H and ^13^C NMR, see Table 1. It existed in a mixture with compound **4** in the ratio of ~4:1 **(**^1^H and ^13^C NMR values for compound **4** are presented in Table 1). Impurities of other related compounds (less than 5 mol%) and lipid (6–14 mol%) were present.

Compound **3** was identified as bis(2,3-dibromo-4,5-dihydroxylbenzyl) ether, first published by Kurihara and coworkers [29]. ^1^H NMR (600 MHz, CDCl_3_) δ_H_ 5.46 (s, OH), 5.60 (s, OH), 7.16 (s, 1H), 4.61 (s, 1H). ^13^C NMR (600 MHz, CDCl_3_) δ_C_ 113.20 (s, C-3′), 140.60 (s, C-4′), 143.41 (s, C-5′), 115.11 (d, C-6′), 131.67 (s, C-1′), 72.64 (d, C-7′), 113.80 (s, C-2′). Impurities of other related compounds (10 mol%) and lipid (less than 5%) were present. 

Compound **4** was identified as 5,5″-oxybis(methylene)bis(3-bromo-4-(2′,3′-dibromo-4′,5′-dihydroxylbenzyl)benzene-1,2-diol) and not reported previously. ^13^C, ^1^H and HMBC data are presented in Table 1. High resolution MS spectra (Figure S1) and the following NMR spectra are attached in the supplementary material: ^1^H (Figure S2), ^13^C (Figure S3), superimposed HSQC and HMBC (Figure S4). Compound **4** existed in a mixture with compound **2** in the ratio of ~1:1.5. Impurities of lipid (20–40 mol%) were present. 

### 3.6. Preparation of Solutions

A 20 mg/mL stock solution in DMSO was prepared for each compound. Working solutions were diluted in RPMI-1640 for the cell viability assay, while in MilliQ water for the remaining assays. Final test concentrations for the cell viability assay were 5, 25 and 50 μg/mL. In the ORAC assay, final test concentrations were 1, 5, 10, 25 and 50 μg/mL, while they were 1, 5, 10, 20 and 50 μg/mL for CAA and CLPAA. An exception was compound **3**, where 30 μg/mL was the highest concentration in the antioxidant assays. 

### 3.7. Cell Culture and Seeding

MRC-5 (human fetal lung; ATCC, CCL-171™) cells were grown in RPMI-1640 and HepG2 (human hepatocellular liver carcinoma; ATCC HB-8065™) in MEM-Earle’s medium (F0325); both media were supplemented with fetal bovine serum (10%, S0115), non-essential amino acids (1%, K0293), sodium pyruvate (1 mM, L0473), l-alanyl-l-glutamine (2 mM, K0302) and gensumycin (10 μg/mL, A2712) and incubated at 37 °C with 5% CO_2_. All incubations were performed at 37 °C with 5% CO_2_. Media and supplements were all from Biochrom (Berlin, Germany). 

### 3.8. Cytotoxicity

Cell viability was determined by a colorimetric [3-(4,5-dimethylthiazol-2-yl)-5-(3-carboxymethoxyphenyl)-2-(4-sulfophenyl)-2*H*-tetrazolium] (MTS) assay.

MRC-5 cells were seeded in 96-well microtiter plates (Nunc 167008) at a concentration of 2000 cells/well and incubated for 24 h. After the media was removed, RPMI-1640 with 10% FBS, in addition to the compounds in triplicate, were added. The plate was then incubated for 72 h at 37 °C in a humidified atmosphere of 5% CO_2_. At the end of the exposure time, CellTiter 96^®^ AQ_ueous_ One Solution Reagent (Promega, Madison, WI, USA) was added to each well and the plate incubated for 1 h. Absorbance was measured at 485 nm in a DTX 880 Multimode Detector (Beckman Coulter, CA, USA). 

Cells in RPMI-1640 medium were used as negative control and cells treated with Triton^®^ X-100 (Sigma-Aldrich) reagent as positive control. Relative cell survival was determined by using the measured optical density (OD), calculated as: cell survival (%) = (OD treated well − OD positive control)/(OD negative control − OD positive control) × 100. 

### 3.9. Antioxidant Assay for Oxygen Radical Absorbance Capacity (ORAC)

The assay was performed based on the procedure of Huang *et al* [19]. 

The method was carried out in black 96-well plates (Nunc 7350004). Fluorescence was recorded using a 1420 Victor3 Plate Reader (Perkin Elmer, MA, USA, with the software, Workout 2.0, from Dazaq Solutions Ltd.) at 37 °C. The compounds, and Trolox (Sigma-Aldrich, 238813) at final concentrations of 0–17 μM included to construct a standard curve, were added in duplicate, followed by the addition of fluorescein (Fluka, 46960) (final concentration of 55 nM). Quercetin dehydrate (Sigma-Aldrich, 200595) and luteolin (Cayman #10004161) (final concentrations of 1, 5, 10, 25 and 50 μg/mL) were used as antioxidant controls. After a 15-minute incubation, AAPH (2,2′-azobis(2-methylpropioanamidine) dihydrochloride; Sigma-Aldrich, 440914) (final concentration of 16 mM) was added to each sample. Fluorescence was recorded 25 times with a 70-second delay between the repeats. Excitation (486 nm) and emission (520 nm) were used. Phosphate buffer (PB) was used as a blank for the 0 μM Trolox sample. The area under the curve (AUC) was calculated with the AUC_blanc_ values subtracted. A standard curve was made using the Trolox values, and Trolox equivalents of the samples were calculated. Results were expressed as μM Trolox equivalents (TE). Total reaction volume was 210 µL (where 25 µL was the volume of the test compound). All reagents were dissolved in 75 nM PB (pH 7.4), and the incubations were at 37 °C. 

### 3.10. Antioxidant Assay for Cellular Antioxidant Activity (CAA)

The assay was performed based on the procedure of Wolfe and Liu (2007) [22]. 

The method was carried out in black 96-well plates (#3603, Corning, NY, USA) with an optical bottom. Fluorescence was recorded using the same plate reader, software and temperature as for the ORAC assay. HepG2 cells were seeded at a density of approximately 80,000 cells/well and incubated overnight. Cells were then incubated with DCFH-DA (2′7′-dichlorofluorescin diacetate; Fluka, 35847) (final concentration of 25 μM) and the test compounds in duplicate for 1 h. Quercetin and luteolin (final concentrations of 1, 5, 10, 20 and 50 (or 30) μg/mL) were used as antioxidant controls. After incubation, Hank’s saline solution without phenol red (Biochrom, BCHRL2035) supplemented with AAPH (final concentration of 600 μM) was added to all the wells, except for the negative control. To these wells was added Hank’s saline solution without AAPH. The plate was immediately placed in the plate reader and fluorescence recorded; excitation of 485 nm and emission of 520 nm were used. The plate was incubated for 1 h before a second reading. Cells were washed with PBS between the addition of new reagents. The total reaction volume was 100 μL (where 20 μL was the volume of the test compound). The incubations were at 37 °C in a humidified atmosphere of 5% CO_2_. 

Cells in MEM Earle’s medium were used as negative control and cells treated with AAPH as positive control. The oxidative degeneration of DCFH-DA to DCF was calculated as: activity (%) = (fluorescence treated well − fluorescence positive control)/(fluorescence negative control − fluorescence positive control) × 100. Fluorescence value: fluorescence reading 2 − fluorescence reading 1. Inhibition (%) was calculated as: 100% − activation%. The results are presented as relative values compared to the positive and negative controls, with and without AAPH, respectively.

### 3.11. Cellular Lipid Peroxidation Antioxidant Activity (CLPAA) Assay

The assay was performed based on the procedure of Pap *et al.* [23].

The method was carried out in the same plates as in the CAA assay. Fluorescence was recorded using the same plate reader, software and temperature as for the ORAC and CAA assays. HepG2 cells were seeded and incubated overnight, as described in the CAA assay. The cells were labelled with 5 µM C_11_-BODIPY (#D3861, Invitrogen, Eugene, OR, USA) for 30 min, following the addition of compounds at various concentrations and incubating for 1 h. Quercetin and luteolin (final concentrations of 1, 5, 10, 20 and 50 (or 30) μg/mL) were used as antioxidant controls. Cumene hydroperoxide (cumOOH, 247502, Sigma-Aldrich, St. Louis, MO, USA) (at a final concentration of 50 µM) in Hank’s saline solution without phenol red was added to initiate lipid peroxidation. The addition was done to all the wells, except the ones for negative control, to which were added Hank’s saline solution without phenol red and without cumOOH. The plate was immediately placed in the plate reader. Both red [590 (excitation)/632 (emission)] and green [485 (excitation)/520 (emission)] fluorescence was recorded during ~1 h at 37 °C. Cells were washed with PBS between additions of new reagents. The total reaction volume was 100 μL (where 20 μL was the volume of the test compound). All incubations were carried out at 37 °C in a humidified atmosphere of 5% CO_2_.

Cells in MEM Earle’s medium were used as negative control and cells treated with cumOOH as positive control. The oxidative degeneration of C_11_-BODIPY was calculated based on the increase of green fluorescence as: activity (%) = (fluorescence treated well − fluorescence positive control)/(fluorescence negative control − fluore scence positive control) × 100. Inhibition (%) was calculated as: 100% − activation%. The results are presented as relative values compared to the positive and negative controls, with and without cumOOH, respectively.

## 4. Conclusions

Cellular antioxidant effects for the bromophenol, 2,2′,3-tribromo-3′,4,4′,5-tetrahydroxy-6′-hydroxymethyl-diphenylmethane (compound **2**), have been reported for the first time. The fact that the compound was active in the CAA assay demonstrates that it enters cells, and as it was also active in the CLPAA assay, it shows the ability to decrease lipid peroxidation in cell membranes. Compound **2** was a better antioxidant than luteolin in both cellular assays and of quercetin in the CLPAA assay, and it was active well below the cytotoxic concentration in all assays. 

The structure of compound **4** and its activity in biological assays is reported for the first time, and compared to quercetin and luteolin, it showed a weak antioxidant activity at 50 μg/mL in all the assays (Figure 1, Figure 2, Figure 3). The possibility of being an artefact of the extraction and purification process has been discussed. 

Only a few studies have been reported on structure-activity relationships (SARs) for BPs, and the ones published are all from *in vitro* biochemical assays [3]. Hydroxyl substitution, a 1,4-dihydroxy arrangement, conjugation and bromination are all factors believed to influence the antioxidant activity of BPs in these assays. Results show that, more extensively, hydroxyl substitution increases antioxidant activity of BPs in DPPH radical scavenging assays [3,6,7,8,30,31]. This corresponds well with the results from our cellular assays, where compound **2**, having one hydroxyl substituent more, had a greater antioxidant activity than compound **3**. However, compound **4** contained three hydroxyl substituents more than compound **2**, but was found to be less active than the latter. An explanation for this might be that compound **4** is a larger molecule, which can influence the cellular uptake. The 1,4-dihydroxy arrangement has been reported to be very suitable for antioxidant activity, and compound **2** resembles such an arrangement, having a hydroxyl and a hydroxymethyl in *para* position to each other [3,32]. The degree of conjugation is proposed as an important factor in increasing the antioxidant activity of BPs [3]. Nevertheless, compound **2** does not have a higher degree of conjugation, so this does not explain the results in the present study. Bromination of BPs has also been investigated in DPPH radical scavenging assays. The antioxidant activity was reported both as decreasing, when compared to corresponding debrominated compounds, and as slightly increasing, when compared to corresponding chlorinated compounds. Liu and co-workers, therefore, concluded that bromination seemed to be of little importance for the antioxidant activity of BPs [3,9,31]. Halogenation of polar molecules is a known way to increase their lipophilicity and, consequently, increase their bioavailability [33]. Thus, although bromination was found not to be of importance for antioxidant activity in biochemical assays, it might influence the bioavailability of compounds. To be active in cellular assays, the compounds have to penetrate the cell membrane, and the ability to do so is influenced by their size, solubility, polarity and charge. We believe that the poor activity of compound **4** in the cellular assays is due to its larger size compared to compound **2**.

This present identification of cellular antioxidant activity of structurally related BPs can be used as a starting point for SAR studies, which are more relevant to *in vivo* conditions, taking into account factors like bioactivity and membrane permeability. The cellular assays can be used to, at an early stage, exclude compounds lacking the ability of penetrating membranes and, hence, do not have an effect *in vivo*. 

## Supplementary Files

Supplementary File 1 Supplementary (PDF, 322 KB)

## References

[1] El Gamal A.A. (2010). Biological importance of marine algae. Saudi Pharm. J..

[2] Whitfield F.B., Helidoniotis F., Shaw K.J., Svoronos D. (1999). Distribution of bromophenols in species of marine algae from eastern Australia. J. Agric. Food Chem..

[3] Liu M., Hansen P.E., Lin X. (2011). Bromophenols in marine algae and their bioactivities. Mar. Drugs.

[4] Flodin C., Helidoniotis F., Whitfield F.B. (1999). Seasonal variation in bromophenol content and bromoperoxidase activity in *Ulva lactuca*. Phytochemistry.

[5] Li K., Li X.M., Gloer J.B., Wang B.G. (2011). Isolation, characterization, and antioxidant activity of bromophenols of the marine red alga *Rhodomela confervoides*. J. Agric. Food Chem..

[6] Duan X.-J., Li X.-M., Wang B.-G. (2007). Highly brominated mono- and bis-phenols from the marine red alga *Symphyocladia latiuscula* with radical-scavenging activity. J. Nat. Prod..

[7] Li K., Li X.-M., Ji N.-Y., Wang B.-G. (2007). Natural bromophenols from the marine red alga *Polysiphonia urceolata* (Rhodomelaceae): Structural elucidation and DPPH radical-scavenging activity. Bioorganic Med. Chem..

[8] Li K., Li X.-M., Ji N.-Y., Wang B.-G. (2008). Bromophenols from the marine red alga *Polysiphonia urceolata* with DPPH radical scavenging activity. J. Nat. Prod..

[9] Lee J.-H., Lee T.-K., Kang R.-S., Shin H.-J., Lee H.-S. (2007). The *in vitro* antioxidant activities of the bromophenols from the red alga *Tichocarpus crinitus* and phenolic derivatives. J. Korean Magn. Reson. Soc..

[10] Saito T., Ando Y. (1955). Bromine compounds in sea-weeds. I. On a bromophenolic compound obtained from the red algae, *Polysiphonia Morrowii Harv*. Nippon Kagaku Zassi.

[11] Zhao J., Fan X., Wang S., Li S., Shang S., Yang Y., Xu N., Lü Y., Shi J. (2004). Bromophenol derivatives from the red alga *Rhodomela confervoides*. J. Nat. Prod..

[12] Guiry M.D., Guiry G.M. http://www.algaebase.org.

[13] Zubia M., Fabre M.S., Kerjean V., Deslandes E. (2009). Antioxidant and cytotoxic activities of some red algae (Rhodophyta) from Brittany coasts (France). Bot. Mar..

[14] Devasagayam T., Tilak J., Boloor K., Sane K.S. (2004). Free radicals and antioxidants in human health: Current status and future prospects. J. Assoc. Physicians India.

[15] Wu D.F., Cederbaum A.I. (2003). Alcohol, oxidative stress and free radical damage. Alcohol Res. Health.

[16] Mallick N., Mohn F.H. (2000). Reactive oxygen species: Response of algal cells. J. Plant Physiol..

[17] Kamalakar G., Komura K., Kubota Y., Sugi Y. (2006). Friedel–Crafts benzylation of aromatics with benzyl alcohols catalyzed by heteropoly acids supported on mesoporous silica. J. Chem. Technol. Biotechnol..

[18] Huang D., Ou B., Prior R.L. (2005). The Chemistry behind Antioxidant Capacity Assays. J. Agric. Food Chem..

[19] Huang D., Ou B., Hampsch-Woodill M., Flanagan J.A., Prior R.L. (2002). High-Throughput assay of oxygen radical absorbance capacity (orac) using a multichannel liquid handling system coupled with a microplate fluorescence reader in 96-well format. J. Agric. Food Chem..

[20] Wolfe K.L., Liu R.H. (2008). Structure–Activity relationships of flavonoids in the cellular antioxidant activity assay. J. Agric. Food Chem..

[21] Hofer T., Eriksen T.E., Hansen E., Varmedal I., Jensen I.-J., Hammer-Andersen J., Olsen R.L., Basu S., Wiklund L. (2011). Cellular and Chemical Assays for Discovery of Novel Antioxidants in Marine Organisms. Studies on Experimental Models.

[22] Wolfe K.L., Liu R.H. (2007). Cellular antioxidant activity (CAA) assay for assessing antioxidants, foods, and dietary supplements. J. Agric. Food Chem..

[23] Pap E.H.W., Drummen G.P.C., Winter V.J., Kooij T.W.A., Rijken P., Wirtz K.W.A., Op den Kamp J.A.F., Hage W.J., Post J.A. (1999). Ratio-fluorescence microscopy of lipid oxidation in living cells using C11-BODIPY581/591. FEBS Lett..

[24] Drummen G.P.C., van Liebergen L.C.M., Op den Kamp J.A.F., Post J.A. (2002). C11-BODIPY581/591, an oxidation-sensitive fluorescent lipid peroxidation probe: (micro)Spectroscopic characterization and validation of methodology. Free Radic. Biol. Med..

[25] Liu R.H., Finley J. (2005). Potential cell culture models for antioxidant research. J. Agric. Food Chem..

[26] Fan X., Xu N.J., Shi J.G. (2003). Two new bromophenols from red alga *Rhodomela confervoides*. Chinese Chem. Lett..

[27] Gottlieb H.E., Kotlyar V., Nudelman A. (1997). NMR Chemical Shifts of Common Laboratory Solvents as Trace Impurities. J. Org. Chem..

[28] Katsui N., Suzuki Y., Kitamura S., Irie T. (1967). 5,6-Dibromoprotocatechualdehyde and 2,3-dibromo-4, 5-dihydroxybenzyl methyl ether: New dibromophenols from *Rhodomela larix*. Tetrahedron.

[29] Kurihara H., Mitani T., Kawabata J., Takahashi K. (1999). Two new bromophenols from the red alga *Odonthalia corymbifera*. J. Nat. Prod..

[30] Choi J.S., Park H.J., Jung H.A., Chung H.Y., Jung J.H., Choi W.C. (2000). A cyclohexanonyl bromophenol from the red alga *Symphyocladia latiuscula*. J. Nat. Prod..

[31] Zhao W., Feng X., Ban S., Lin W., Li Q. (2010). Synthesis and biological activity of halophenols as potent antioxidant and cytoprotective agents. Bioorg. Med. Chemi. Lett..

[32] Chen L., Fang Y., Zhu T., Gu Q., Zhu W. (2008). Gentisyl alcohol derivatives from the marine-derived fungus *Penicillium terrestre*. J. Nat. Prod..

[33] Gentry C.L., Egleton R.D., Gillespie T., Abbruscato T.J., Bechowski H.B., Hruby V.J., Davis T.P. (1999). The effect of halogenation on blood-brain barrier permeability of a novel peptide drug. Peptides.

