# Supplementary material for: Cellular Antioxidant Effect of Four Bromophenols from the Red Algae, Vertebrata lanosa
Source: Mar Drugs. 2013 Aug 5;11(8):2769–84. doi: 10.3390/md11082769 (PMC3766864; doi:10.3390/md11082769)

Supplementary Materials

Figure S1. High resolution MS spectra of compound **4**.

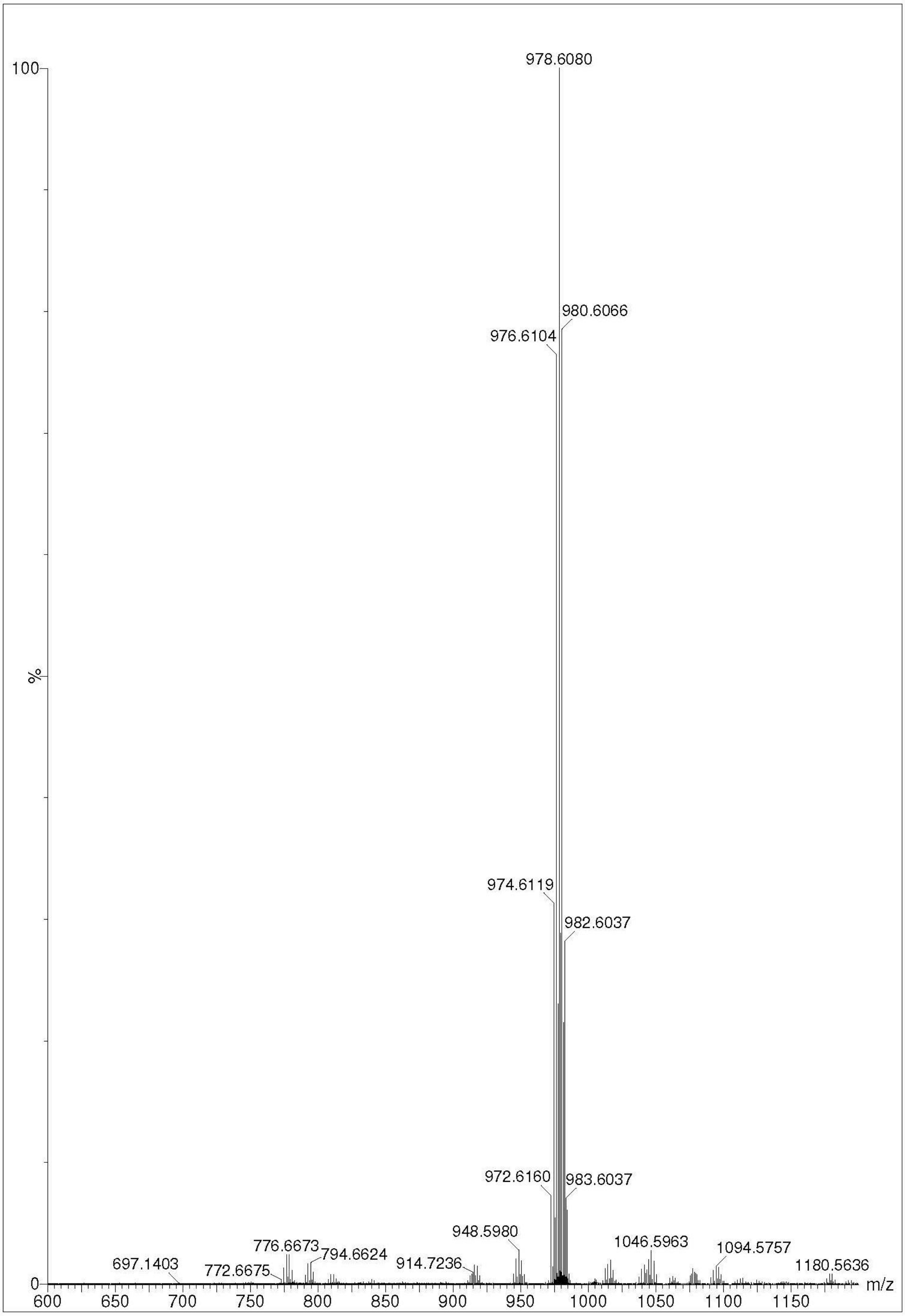

Figure S2. Proton NMR spectrum of **2** (black annotations from Scheme 1) and **4** (blue annotations) in methanol-d_4 with Wet suppression on the water resonance.

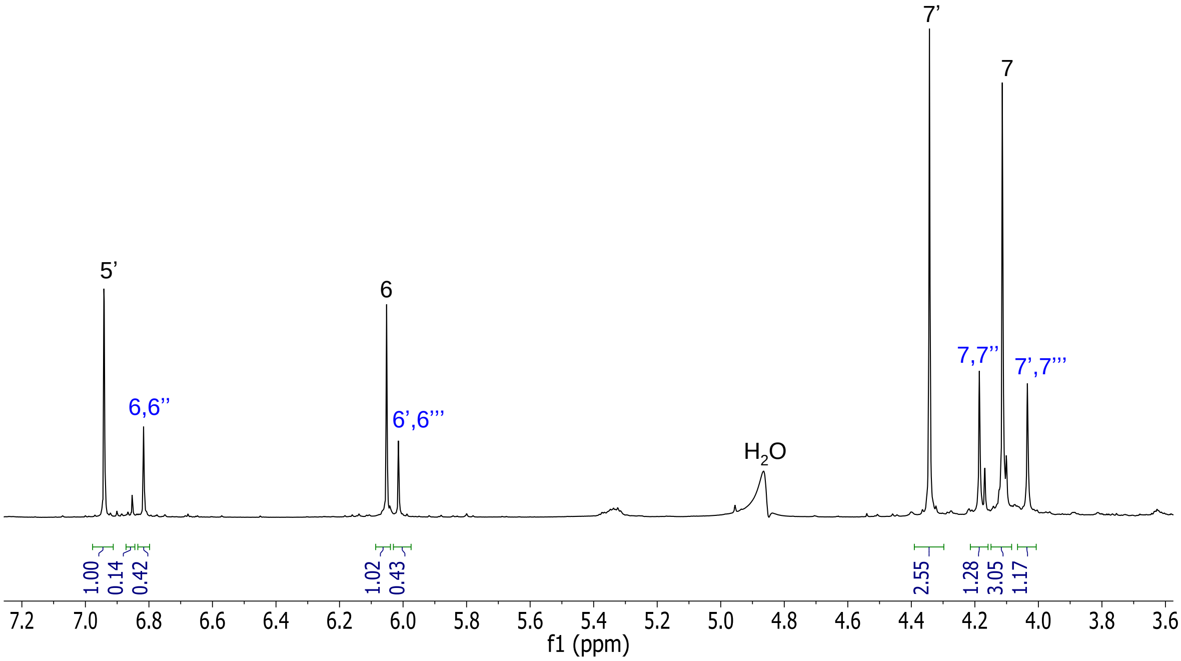

Figure S3. Proton decoupled Carbon NMR spectrum of **2** (black annotations from Scheme 1) and **4** (blue annotations).

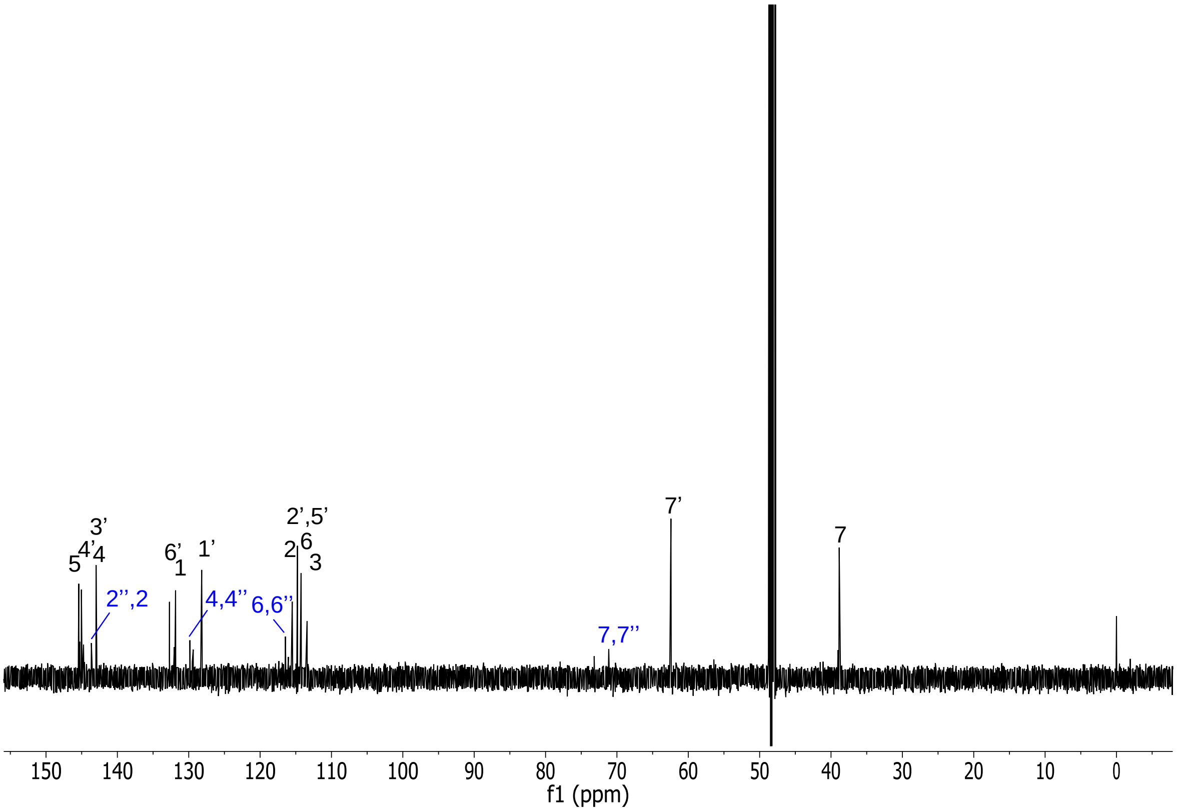

Figure S4. Superimposed gradient selected HSQC and HMBC spectra of **2** (black annotations from Scheme 1) and **4** (blue annotations).

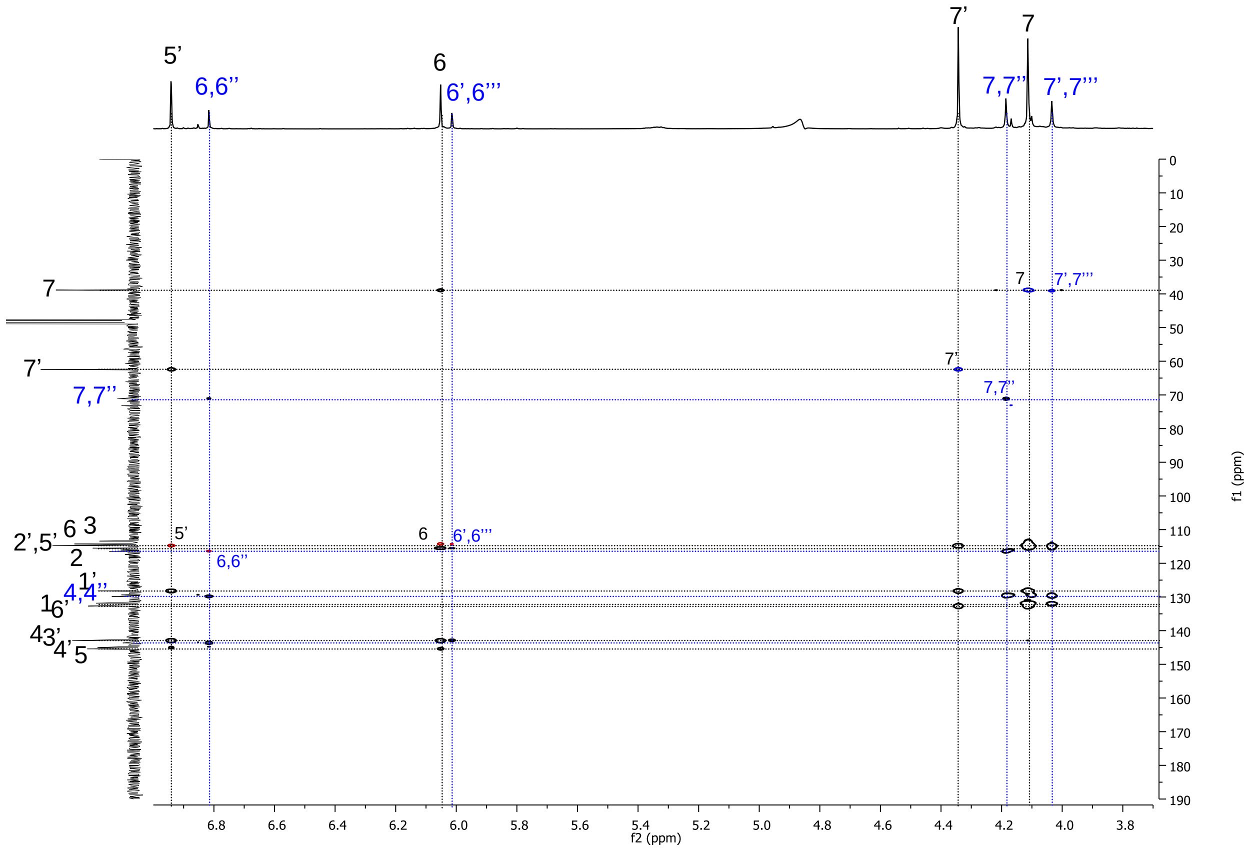

Figure S5. Superimposed gradient selected HSQC and HMBC spectra zooming in on the overlapped $^{1}J_{CH}$ (blue) and $^{3}J_{CH}$ (black) correlations of the two symmetric methylenes at position 6 for compound **4**, distinctive for the dimerization.

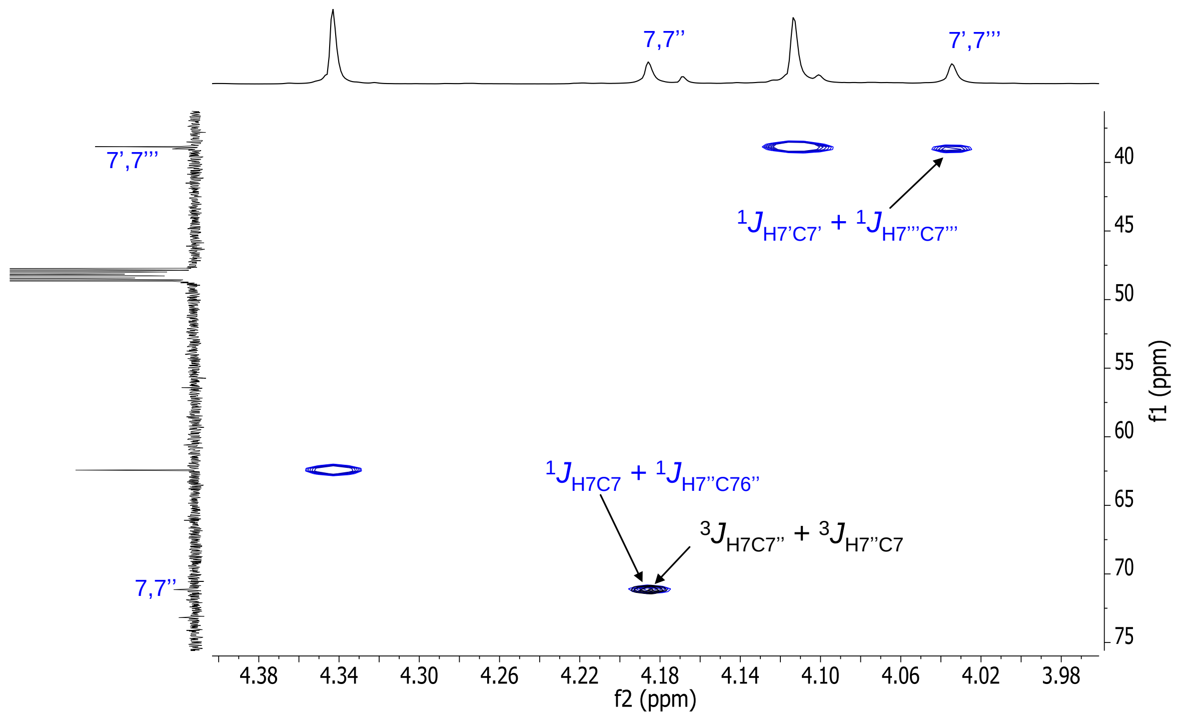

Supplement: Supplementary File 1 — Supplementary (PDF, 322 KB) [file marinedrugs-11-02769-s001.pdf]
